# Hyper-FET’s Phase-Transition-Materials Design Guidelines for Ultra-Low Power Applications at 3 nm Technology Node

**DOI:** 10.3390/nano12224096

**Published:** 2022-11-21

**Authors:** Hanggyo Jung, Jeesoo Chang, Changhyun Yoo, Jooyoung Oh, Sumin Choi, Juyeong Song, Jongwook Jeon

**Affiliations:** 1 Department of Electrical and Electronics Engineering, Konkuk University, Seoul 05029, Republic of Korea; 2 Data and Information Tech. (DIT) Center, Samsung Electronics, Hwasung-Si 18448, Republic of Korea

**Keywords:** Boltzmann limit, low power application, phase-transition-materials (PTM), steep switching device, hybrid-phase-transition FETs (hyper-FET)

## Abstract

In this work, a hybrid-phase transition field-effects-transistor (hyper-FET) integrated with phase-transition materials (PTM) and a multi-nanosheet FET (mNS-FET) at the 3 nm technology node were analyzed at the device and circuit level. Through this, a benchmark was performed for presenting device design guidelines and for using ultra-low-power applications. We present an optimization flow considering hyper-FET characteristics at the device and circuit level, and analyze hyper-FET performance according to the phase transition time (TT) and baseline-FET off-leakage current (I_OFF_) variations of the PTM. As a result of inverter ring oscillator (INV RO) circuit analysis, the optimized hyper-FET increases speed by +8.74% and reduces power consumption by −16.55%, with I_OFF_ = 5 nA of baseline-FET and PTM TT = 50 ps compared to the conventional mNS-FET in the ultra-low-power region. As a result of SRAM circuit analysis, the read static noise margin is improved by 43.9%, and static power is reduced by 58.6% in the near-threshold voltage region when the PTM is connected to the pull-down transistor source terminal of 6T SRAM for high density. This is achieved at 41% read current penalty.

## 1. Introduction

Silicon-based metal–oxide–semiconductor field-effect transistors (MOSFETs) are being scaled down to sub-5 nm, and a multi-nanosheet FET (mNS-FET) is expected to be used in the 3 nm technical node. Although successful scaling down technology is being developed, increase in power density has become an issue due to difficulty in reducing supply voltage (V_DD_). Recently, research on overcoming these limitations by achieving a sub-threshold swing (SS) ≤60 mV/dec through devices such as negative capacitance FET (NC-FET) [1] and tunneling FET (T-FET) [2] using nanomaterials is being actively conducted. In addition, a Hybrid-Phase Transition Field-Effects-Transistor (hyper-FET) [3] device utilizing the steep switching characteristic of nano-scale Phase-Transition Materials (PTM) [4,5] has been proposed, and various material and process prospective studies are actively being conducted. Recently, a study analyzed for the first time in terms of the operation characteristics of a hyper-FET device and circuit using a fin-FET of a 14 nm technology node as a baseline FET was published by Aziz, A. et al. in [6,7]. However, there are insufficient studies on the electrical characteristics of a hyper-FET upon scaling down to the latest node, such as a 3 nm technology node, and targeting of the PTM characteristics for hyper-FET device design.

In this work, following the approach of Aziz, A. et al., we focused on PTM device design for hyper-FET implementation, based on a 3 nm technology node using a mNS-FET with various threshold voltage (V_th_) characteristics. We propose an optimization method in consideration of device and circuit operation for optimizing the key electrical characteristics of baseline-FET and PTM constituting hyper-FET. This is a proposed flow based on a systematic analysis of previously published research results [6,7]. Hyper-FET characteristics were secured according to I_OFF_ of baseline-FET and variation of the PTM’s transition time (TT) through the systematic optimization process, and benchmarks were performed in logic INV RO and SRAM circuits with conventional CMOS devices. Since an INV RO is a circuit that represents the power and speed characteristics of numerous logic standard cell circuits, such as NAND, NOR, and XOR, the benchmark through an INV RO can be considered to have the characteristics of the entire logic circuit. Also, hyper-FET design guidelines for further circuit characteristic optimization at the 3 nm tech. node are presented

Chapter 2 of this paper describes the device and circuit simulation environment used in this work, Chapter 3 presents the hyper-FET optimization process and the logic circuit level (INV RO, SRAM) results of benchmark performance with conventional CMOS devices, and Chapter 4 presents the conclusion of this paper.

## 2. Simulation Environment for Hyper-FET Device and Circuit Co-Analysis

**Figure 1:**
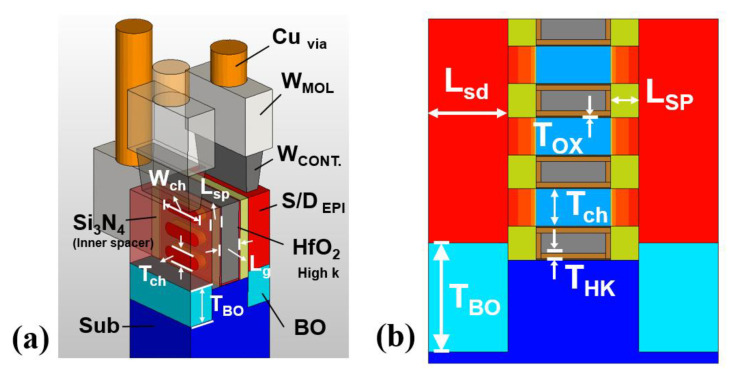
(**a**) Three-dimensional structure and (**b**) cross-section view of the mNS-FET used in this work.

In this work, a lateral multi-nanosheet field-effect transistor (LmNS-FET) using multiple multi-nanosheet channels placed in the lateral direction in the 3 nm technology node dimension, was used as an FEOL device. The number of nanosheet channels used is three, and the main device dimensions of the 3 nm technology node, such as contact-poly-pitch (CPP) and gate length (L_g_), were set concerning IRDS 2020 [8] as shown in Figure 1. Table 1 summarizes the results.

**Figure 2:**
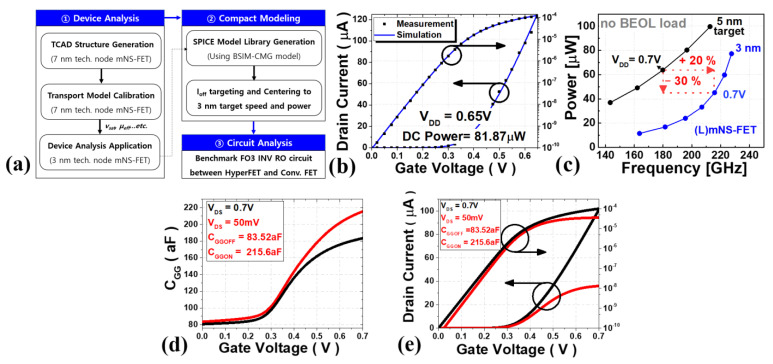
(**a**) The overall workflow for the analysis of mNS-FET-based device and circuit analysis, (**b**) calibration results of transport model parameters in TCAD software with measured data, and (**c**) the FO3 INV RO simulation result after I_OFF_ targeting and centering to the 3 nm technology node target, (**d**,**e**) C_GG_ versus V_GS_ & I_DS_ versus V_GS_ characteristics in this model.

The electrical characteristics of the mNS-FET were simulated using Synopsys’ Sentarurus^TM^ ver. S-2021.06-SP1, a three-dimensional TCAD software, and the model used in this case was used by adding the following models to the drift-diffusion carrier transport model. The density gradient quantization model (eQuantum Potential) was included to describe the quantum confinement effect, and the mobility model (phumob/high field saturation/Enormal) was utilized to consider the quantum effect, Coulomb scattering, and interfacial surface roughness scattering. The Lombardi mobility model was included to calculate the mobility degradation by remote phonon and Coulomb scatterings at the channel and insulator interface. A thin-layer mobility model was included to account for the thin channel thickness. The measured electrical characteristics of the mNS-FET manufactured by hardware in the 7 nm dimension and the TCAD model parameters were calibrated to increase the accuracy of the model. A model library that accurately describes the current-voltage (I-V) and capacitance-voltage (C-V) characteristics extracted through TCAD simulation were then created using the industry-standard model BSIM-CMG. Next, to have the industry’s standard 3 nm model circuit characteristics, the generated model library was centered to satisfy the target speed and power of the INV RO with fan-out 3 (FO3), a benchmark circuit, to produce the final model library as summarized in Figure 2.

**Figure 3:**
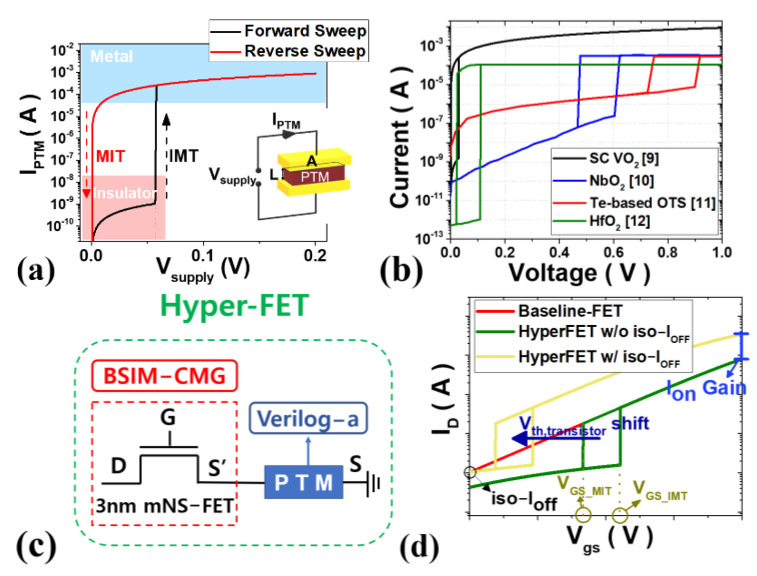
(**a**) I-V characteristics of PTM; (**b**) Some examples of PTM; (**c**) Compact modeling structure of hyper-FET; (**d**) Transfer curve of baseline-FET and hyper-FET before and after I_OFF_ targeting.

Next, the PTM compact model was developed using Verilog-A to describe the I-V characteristics according to the voltage applied to the PTM as shown in Figure 3a. The developed PTM have a low resistivity ρ_MET_ in the metal phase and a large resistivity ρ_INS_ in the insulator phase, and the current determined by the voltage applied across the PTM device is compared with the critical current density (J_C_) at which the phase transition occurs. Such an operation in which either an insulator-metal transition (IMT) or a metal-insulator transition (MIT) occurs through the circuit simulator can be implemented. This model reflects the characteristics of PTMs such as single crystalline VO_2_ [9], NbO_2_ [10], Te-based OTS [11] and HfO_2_ [12], such as in Figure 3b, and the key parameters are summarized in Table 2. In addition, an RC-delay circuit is implemented inside Verilog-A to describe transition time (TT) [13], which has a very important effect on the circuit’s dynamic operation characteristics. The dimension of the PTM, the PTM area (A_PTM_) and the PTM length (L_PTM_) are 30 nm × 15 nm (metal pitch × source/drain contact length) and 20 nm, respectively, considering the process design rule of the 3 nm tech. node.

As shown in Figure 3c, the compact model of the hyper-FET with the PTM connected in series to the source terminal of the baseline-FET has the transfer characteristics as shown in Figure 3d. Gate voltage conditions in which an IMT and MIT occur are named V_GS_IMT_ and V_GS_MIT_, respectively. Assuming that the V_th_-shift of the baseline FET is possible through a process such as work-function engineering, the hyper-FET is expected to improve on-current compared to the conventional mNS-FET under the iso-I_OFF_ condition.

## 3. Hyper-FET Simulation Results and Discussions

For hyper-FET optimization, it is necessary to determine the electrical characteristics of each device under systematical analysis since there is a very close relationship between the composing baseline-FET and the electrical characteristics of the PTM. In this work, the key parameters of the PTM (ρ_INS_, ρ_MET_, J_C_IMT_, J_C_MIT_) were determined through the circuit characteristics according to the switching time characteristics of the PTM and the variation in baseline-FET I_OFF_ characteristics, which were not considered in previous studies. Through this, hyper-FET design optimization was performed, and circuit-level benchmarks were performed with conventional 3 nm tech. mNS-FET.

### 3.1. Hyper-FET Design Optimization Flow

**Figure 4:**
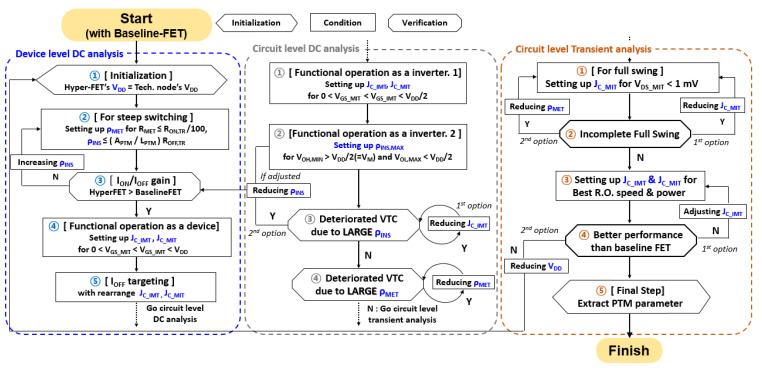
An optimal PTMs parameter extraction flow chart.

For optimal design of hyper-FET devices, device level DC (DD), circuit level DC (CD), and circuit level transient (CT) analysis are essential, as shown in Figure 4. In this subchapter, the systematic optimization process used in this work is described.

#### 3.1.1. Device Level DC Analysis

**Figure 5:**
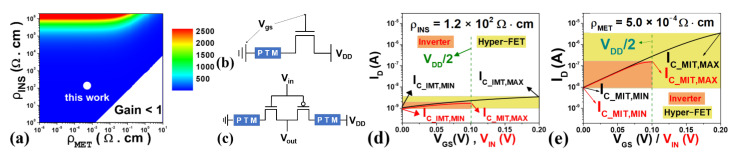
(**a**) I_ON_/I_OFF_ gain mapping of hyper-FET compared to baseline-FET; (**b**,**c**) schematic of the hyper-FET and the hyper-FET-based inverter; (**d**,**e**) I_C_IMT_ & I_C_MIT_ ranges for proper operation in the I-V curves of (**b**) and (**c**) (yellow box & black text for (**b**), orange box & red text for (**c**)).

As he initial setting of the hyper-FET’s V_DD_ is used as the supply voltage of the baseline-FET, the conditions under which the resistance of the PTM (R_PTM_) for the hyper-FET have steep slope characteristics are discussed. In order to prevent the PTM’s resistance of the metal state (R_MET_) from inhibiting the on-current of the hyper-FET, the ρ_MET_ is determined to be at least 100 times smaller than the resistance of the transistor in the on state (R_ON,TR_ at V_GS_ = V_DD_, V_DS_ = V_DD_). Then, the resistance of the insulator state of the PTM (R_INS_) is determined through Figure 5a, a graph where Hyper-FET I_ON_/I_OFF_ is mapped according to ρ_INS_ and ρ_MET_ made with reference to [6]. The gain increases in proportion to ρ_INS_, but circuit operation may become difficult if too large, as shown in CD-3. Therefore, the ρ_INS_ is set so that R_INS_ is less than the resistance of the transistor in the off state (R_OFF,TR_ at V_GS_ = 0 V, V_DS_ = V_DD_), and I_ON_/I_OFF_ gain is checked compared to the baseline-FET. Then, J_C_IMT_&J_C_MIT_ was designed to include I_C_IMT_&I_C_MIT_ in the yellow box of Figure 5d,e, made with reference to [6], so that hyper-FET, as shown in Figure 5b, transitions within the given supply voltage range.

**Figure 6:**
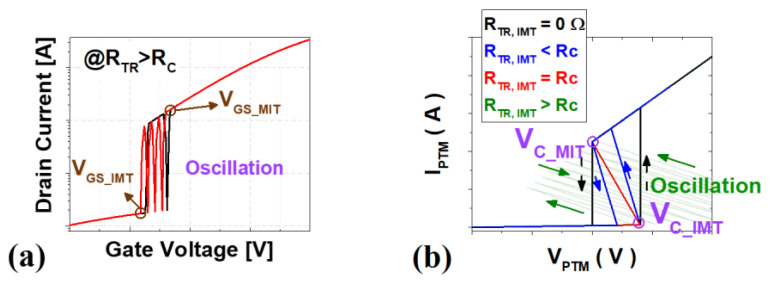
(**a**) The hyper-FET transfer curve with negative hysteresis (**b**) I-V characteristics of PTMs according to R_TR_.

Furthermore, it is necessary to satisfy the condition of V_GS_IMT_ > V_GS_MIT_ to avoid negative hysteresis [14] in which the current of the hyper-FET oscillates, as shown in Figure 6a. This phenomenon can be understood through Figure 6b, which is a graph of voltage biased to the PTM (V_PTM_) and the current graph according to V_GS_. As V_GS_ increases, V_PTM_ increases, and when V_PTM_ reaches critical voltage for an IMT (V_C_IMT_), negative differential resistance (NDR) occurs, in which V_PTM_ decreases and current increases due to the lowered resistance. If the resistance of the transistor during transition (R_TR_) is less than or equal to a critical resistance (R_C_), the PTMs make hysteretic switching. On the other hand, because of the large R_TR_, the relatively lower V_PTM_ is not sufficient to keep the PTM in a metal state, so MIT occurs again, and it repeats and oscillates like a green line, which is called negative hysteresis. If R_TR_ > R_C_, V_GS_ decreases from V_DD_ and V_PTM_ satisfies critical voltage for MIT(V_C_MIT_), the V_PTM_ is too large to keep the PTMs in the resistance state, so they transition back to the metal state and oscillate in the same way. The critical resistance R_C_ is as follows [14]:

(1) $$R_{C}=\frac{\left|V_{C\_\mathrm{IMT}}-V_{C\_\mathrm{MIT}}\right|}{\left|I_{C\_\mathrm{IMT}}-I_{C\_\mathrm{MIT}}\right|}$$

Lastly, I_ON_ gain is obtained by matching the I_OFF_ of hyper-FET with that of the baseline-FET, as shown in Figure 3d (of course, J_C_IMT_ & J_C_MIT_ adjustment is necessary afterwards).

#### 3.1.2. Circuit Level DC Analysis

**Figure 7:**
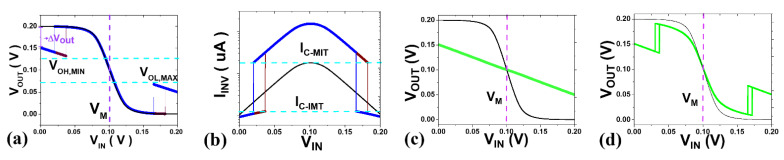
(**a**) Functional hyper-inverter’s VTC; (**b**) Inverter current versus V_IN_ of functional hyper-inverter; (**c**,**d**) Non-functional VTC due to (**c**) large R_INS_; (**d**) large R_MET_.

For functional operation of the logic circuit, the effect of hysteresis, which is not in the existing FET, on the VTC (Voltage-Transfer-Curve) is checked. First, as revealed in previous study [7], if an IMT does not occur before V_M_ (logic threshold voltage) regardless of forwarding sweep or reverse sweep at the VTC in Figure 7a, the inverter current in Figure 7b does not satisfy the IMT condition and switching does not occur within the voltage range. Therefore, design J_C_IMT_ and J_C_MIT_ so that I_C_IMT_ and I_C_MIT_ are included in the orange box in Figure 5d,e are obtained with the inverter in Figure 5c since V_GS_IMT_ & V_GS_MIT_ < V_DD_/2 must be satisfied. In general, the characteristics are improved in proportion to ρ_INS_, so ρ_INS,MAX_ as large as possible is required. However, when this maximum value is exceeded, the high-level output voltage’s minimum (V_OH, MIN_) comes into contact with V_M_, and the VTC collapses, as shown by the green line in Figure 7c. In this case, it is necessary to either reduce J_C_IMT_ with the first option, or reduce ρ_INS_ with the second option. Additionally, if ρ_MET_ is too large, it adversely affects V_OUT_, as shown in Figure 7d. To prevent this, the initial value of ρ_MET_ is set small enough.

#### 3.1.3. Circuit Level Transient Analysis

In DC analysis, V_OUT_ varies as much as ΔV_OUT_, as shown in Figure 7a, and it was revealed in the previous paper [7] that this phenomenon is prevented when the V_DS_MIT_ (V_DS_ at which an MIT occurs) of a hyper-FET is lower than 1 mV. If not, either J_C_MIT_ or ρ_MET_ should be reduced. After verifying that the given values have proper VTC, the performance is evaluated using an INV RO with a fan-out of 3 (FO3) with a hyper-FET. Evaluate various performances by adjusting J_C_IMT_ and J_C_MIT_ under the given ρ_INS_ and ρ_MET_ conditions. V_DD_ scaling down is necessary if the target performance is not obtained because of the TT of the PTM, and has a large influence on speed limiting, which will be dealt with later in Section III-B. In this case, you must start over from DD-1. If the expected performance is satisfied, measure the power and performance and extract the parameters of the PTMs. Table 2 summarizes the key parameters of the PTMs obtained through the above flow when a 3 nm mNS-FET is used as a baseline-FET.

### 3.2. Benchmark with Conventional MOSFET Using INV RO

**Figure 8:**
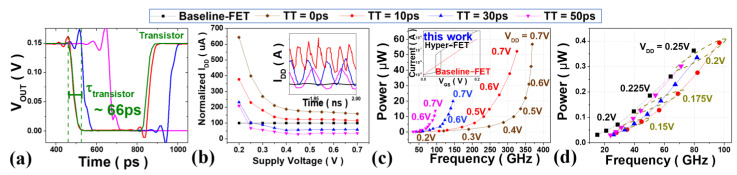
(**a**) INV RO transient waveforms according to the TT of the PTM; (**b**) Normalization of the average hyper-FET’s current (I_DDA_) according to PTMs’ TT to a baseline-FET, and the internal graph shows the transient waveform of the inverter current; (**c**,**d**) Hyper-FET’s circuit characteristics of some PTM’s TT (**a**) at V_DD_ = 0.2~0.7 V (**b**) at V_DD_ ≤ 0.25 V.

First, we discuss the results of studying the optimal characteristics of a hyper-FET for 3 nm tech. mNS-FET according to the TT of the PTM. Figure 8a shows the transient analysis of an INV RO according to the TT. The intrinsic transistor delay (τ_transistor_) required for transition from V_DD_ to GND (or vice versa) is measured as 66 ps. In the case of hyper-FET, the delay is longer than τ_transistor_ due to the TT required for the PTM to change phase. This can be understood through the internal graph of Figure 8b showing the current flowing in the hyper-FET-based INV RO, because the higher the TT of the PTM, the lower the current level. According to Figure 8b, a graph in which the average current of the internal graph is normalized to the average current of the baseline-FET, When the TT is less than 10 ps, the current level is higher than the baseline-FET over all V_DD_ ranges. However, when the TT ≥ 30 ps, a high current level was observed only in the ultra-low power region with a low τ_transistor_ of 0.3 V or less. This indicates that the TT of the PTM suppresses the Hyper-FET from having a higher I_ON_ compared to the baseline-FET at the device level. As the TT of the PTM increases, the circuit performance of the hyper-FET will deteriorate due to the increase in delay and the decrease in the current level. Therefore, in order to use it as a logic device of the hyper-FET, PTM having a TT much smaller than at least τ_transistor_ should be used. Figure 8c shows the circuit characteristics according to the TT of the PTM. As the TT is higher, the current level is lowered and the delay is increased, so the performance and power of the circuit are both reduced. However, this tendency is reduced in the low V_DD_ region, which is relatively smaller than the TT of the PTM due to the increased τ_transistor_ of the transistor.

Figure 8d shows the circuit characteristics with a TT lower than τ_transistor_ in the ultra-low power region (V_DD_ ≤ 0.25 V). The baseline-FET used was I_OFF_ = 5 nA at V_DD_ = 0.7 V, and the value of PTM-3 nm illustrated in Table 2 was used. Table 3 summarizes the operation frequency, active power gain, and supply voltage of the hyper-FET compared to the baseline-FET with V_DD_ = 0.2 V. In all cases of Table 3, the circuit performance was improved even if the supply voltage of the hyper-FET was lower than that of the baseline-FET (V_DD_ = 0.2 V), which is an I_ON_ gain obtained with a steep slope, and the speed was high and the power was decreased due to the low voltage. In case of a TT = 50 ps, compared to a baseline-FET with V_DD_ = 0.2 V, frequency shows a +8.96% and power −16.68% improvement, and their improvement increases as the TT decreases. This shows that at least the TT of the PTMs should be less than 50 ps for the logic device of hyper-FET based on 3 nm tech mNS-FETs.

**Figure 9:**
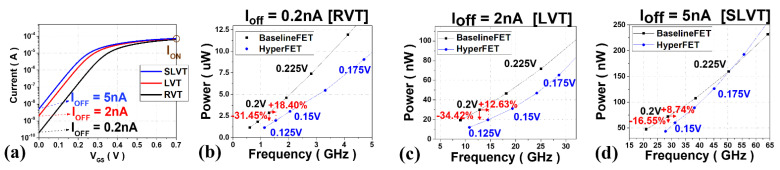
(**a**) Transfer characteristics according to 3 nm tech. mNS-FET’s V_th_ (**b**–**d**) Power and performance evaluation graph of (**b**) RVT (I_OFF_ = 0.2 nA), (**c**) LVT (I_OFF_ = 2 nA), (**d**) SLVT (I_OFF_ = 5 nA).

The following is the result of studying the optimal characteristics of hyper-FET according to V_th_ of the baseline-FET. Figure 9a shows the transfer characteristic of 3 nm tech. mNS-FET according to various threshold voltages, and the RVT (Regular Voltage Threshold), the LVT (Low Voltage Threshold) and the SLVT (Super Low Voltage Threshold) are named for the case where I_OFF_ is 0.2 nA, 2 nA, and 5 nA at V_DD_ = 0.7 V of the baseline-FET, respectively. In addition, Figure 9b–d shows the optimized circuit characteristics in each case of V_DD_ ≤ 0.25 V at TT = 50 ps. Table 4 summarizes the characteristics of the hyper-FET compared to the baseline FET with V_DD_ = 0.2 V. As discussed earlier, the relationship between τ and the TT is significant. The overall current level, which is determined by the V_th_ of the transistor, determines the τ_transistor_, and the higher the V_th_, the freer from the disturbance of the TT. In the case of the RVT device, the hyper-FET shows improvement in frequency by +18.40% and power by −31.45% compared to the baseline-FET. However, as shown in Figure 9c,d, it is observed that as V_th_ decreases, τ_transistor_ approaches the TT, and the amount of performance improvement decreases. In other words, if the designer knows the TT of the PTM, the higher the V_th_, the wider the device’s versatility, and the range of V_th_ that can be optimized is suggested.

**Figure 10:**
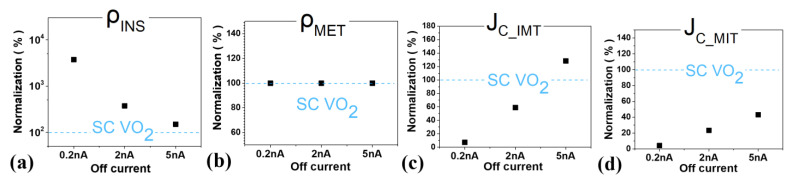
The PTM key parameter trend according to baseline-FET’s I_OFF_ (**a)** ρ_INS_ (**b**) ρ_MET_ (**c**) J_C_IMT_ (**d**) J_C_MIT_.

Figure 10 shows the optimal PTM’s key parameter obtained through the previous flow-based circuit simulation, and shows the tendency according to I_OFF_ by normalizing the parameter of Single Crystalline (SC) VO_2_ [9], one of the most promising candidate PTMs. First, as can be seen from the circuit DC analysis above, the optimal value of ρ_INS_ decreases proportionally as the transistor resistance decreases. Therefore, as the off-current increases, the optimal ρ_INS_ decreases and becomes similar to that of SC VO_2_. On the other hand, the on-current does not change much, so ρ_MET_ set small enough is almost constant. In the case of the critical current density, it shows a tendency to increase as the off-current increases to switch within the current level. In this condition, when I_OFF_ = 5 nA, J_C_IMT_ is 28% larger and J_C_MIT_ is 57% smaller than SC VO_2_. Through this work, if SC VO_2_ is used for hyper-FET utilization of 3 nm tech. mNS-FET, an SLVT device with IOFF = 5 nA is appropriate, and an adjustment of around 60% to the PTM parameter is required.

### 3.3. Benchmark with Conventional MOSFET Using 6T SRAM

**Figure 11:**
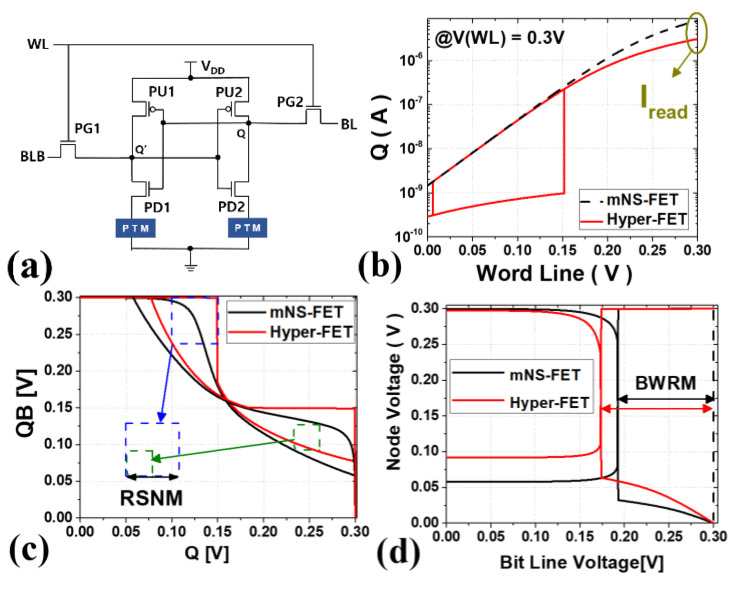
(**a**) The proposed 6T SRAM w/PTM Schematic; (**b**) I_read_; (**c**) RSNM results; (**d**) BWRM results.

In a previous study [15], SRAM topology based on hyper-FET was reported, and a schematic is shown in Figure 11a. The device has a high-density structure with a transistor channel width ratio of pull up: pass gate: pull down = 1:1:1, and the PTM is added to only two pull-down (PD) transistor source terminals out of a total of six transistors, two each. Figure 11b–d shows the results of I_read_ related to read time [16], Read Static Noise Margin RSNM, and Bit-line Write Margin BWRM [17], respectively, compared to mNS-FET 6T SRAM. It is assumed that ‘0’ is stored in Q node and ‘1’ is stored in QB node. In the proposed topology, the PTM in the insulator state of the source of PD1 decreases the strength of PD1 in the read operation and increases the read ability by maintaining the insulator state against noise in Q (or QB). In the write operation, V_Q_ increases due to the PTM in the insulator state connected to PD2, and at the same time, the V_QB_ increases by the PTM in the metal state of PD1 to increase the write ability. Also, the high resistance of the insulator state reduces standby power. As a result of the analysis, the RSNM improved by 43.9%, the BWRM improved by 9.4%, and the static power was reduced by 58.6%. In this case, I_read_ is reduced by 41.4%. Table 5 summarizes the above SRAM index results.

## 4. Conclusions

In this work, we present a systematic design guideline for optimal use of a hyper-FET according to the electrical characteristics of a baseline-FET and the PTM, and, using this, the INV RO and SRAM characteristics according to the PTM’s TT and various characteristics of 3 nm-tech. mNS-FET was evaluated. The presented hyper-FET optimization flow chart allows other researchers to evaluate the optimized logic circuit characteristics of any baseline-FET. Through this, it was confirmed that the circuit characteristics were further improved as the transition time of the PTM was shorter than the intrinsic delay of the transistor. Analysis results show that in the case of 3 nm tech. mNS-FETs, the PTM’s TT should be less than 50 ps in the ultra-low power region (V_DD_ < 0.3 V). In addition, hyper-FET circuit characteristics according to various threshold voltage options of 3 nm tech. mNS-FET related to intrinsic delay were evaluated and the PTM parameter trends were analyzed accordingly. As a result, at the TT = 50 ps of the PTM, the SLVT device with I_OFF_ = 5 nA showed a +8.96% improvement in speed and a −16.68% improvement in power at V_DD_ = 0.15 V. The PTM-3 nm key parameters used in this work are similar to those of Single Crystalline VO_2_ [9]. Also, the 3 nm tech. mNS-FET based 6T SRAM structure with the PTM connected to the pull-down source terminal shows the RSNM improved by 43.9%, the BWRM improved by 9.4%, and the static power was reduced by 58.6%. In this case, I_read_ is reduced by 41.4%. Our systematic optimization flow and the results of logic and memory circuit characteristics not only show the potential for ultra-low power applications of 3 nm technology, but also suggest a direction for PTM optimization of transistors with various characteristics.

## Figures and Tables

**Table 1:** The baseline-FET’s key parameters used in this work.

Parameters	Values
Contacted poly-gate pitch (CPP)	45 nm
Gate length (L_g_)	16 nm
Inner spacer thickness (L_sp_)	6 nm
Channel thickness (T_ch_)	8 nm
Channel width (W_ch_)	30 nm
Channel oxide thickness (T_ox_)	0.3 nm
Channel high-k thickness (T_HK_)	1.1 nm
Bottom oxide thickness (T_BO_)	20 nm
Channel doping	10^17^ cm^−3^
S/D doping	3 × 10^2^ cm^−3^
PTS doping (upper of substrate 1)	1 × 10^19^ cm^−3^
Substrate 2 doping	10^17^ cm^−3^

**Table 2:** PTM’s key parameters & PTM-3 nm values used in this work.

Parameters		Value
ρ_INS_	Resistivity of Insulator state	1.2 × 10^2^ Ω·cm
ρ_MET_	Resistivity of Metal state	5.0 × 10^−4^ Ω·cm
J_C_IMT_	Critical Current Density for IMT	2.4 × 10^2^ A/cm^2^
J_C_MIT_	Critical Current Density for MIT	2.2 × 10^3^ A/cm^2^
TT	PTM Switching Time	50 ps

**Table 3:** Hyper-FET characteristics according to the TT of the PTMs.

I_OFF_ = 5 nA	Baseline-FET (V_DD_ = 0.2 V)	Hyper-FET		
		TT = 50 ps (V_DD_ = 0.15 V)	TT = 30 ps (V_DD_ = 0.1375 V)	TT = 10 ps (V_DD_ = 0.1375 V)
Operating Frequency (GHz)	2.87 × 10^1^ (Ref.)	3.13 × 10^1^ (+8.96%)	3.35 × 10^1^ (+16.89%)	3.50 × 10^1^ (+21.83%)
Active Power (μW)	7.18 × 10^−2^ (Ref.)	5.98 × 10^−2^ (−16.68%)	5.09 × 10^−2^ (−29.10%)	5.08 × 10^−2^ (−29.24%)

**Table 4:** Hyper-FET characteristics according to the baseline-FET’s I_OFF_.

TT = 50 ps	I_OFF_ = 0.2 nA		I_OFF_ = 2 nA		I_OFF_ = 5 nA	
	Baseline-FET (V_DD_ = 0.2 V)	Hyper-FET (V_DD_ = 0.138 V)	Baseline-FET (V_DD_ = 0.2 V)	Hyper-FET (V_DD_ = 0.138 V)	Baseline-FET (V_DD_ = 0.2 V)	Hyper-FET (V_DD_ = 0.15 V)
Operating Frequency (GHz)	1.30 × 10^1^ (Ref.)	1.54 × 10^1^ (+18.40%)	1.29 × 10^1^ (Ref.)	1.45 × 10^1^ (+12.63%)	2.87 × 10^1^ (Ref.)	3.12 × 10^1^ (+8.74%)
Active Power (μW)	2.89 × 10^−2^ (Ref.)	1.98 × 10^−2^ (−31.45%)	3.00 × 10^−2^ (Ref.)	1.97 × 10^−2^ (−34.42%)	7.18 × 10^−2^ (Ref.)	5.99 × 10^−2^ (−16.55%)

**Table 5:** Comparison with 6T SRAM.

	I_read_ (μA)	RSNM (mV)	BWRM (mV)	Static Power (nW)
mNS-FET 6T SRAM	2.68	35.3	107.2	1.28
Hyper-FET 6T SRAM	1.57	50.8	117.3	0.53
Improvement	−41.4%	+43.9%	+9.4%	+58.6%

## Data Availability

Not applicable.
